# Effect of MNCQQ Cells on Migration of Human Dermal Fibroblast in Diabetic Condition

**DOI:** 10.3390/biomedicines10102544

**Published:** 2022-10-12

**Authors:** Sen Jiang, Rie Ito-Hirano, Tsubame Nishikai-Yan Shen, Satoshi Fujimura, Hiroshi Mizuno, Rica Tanaka

**Affiliations:** 1 Division of Regenerative Therapy, Graduate School of Medicine, Juntendo University, 2-1-1, Hongo, Bunkyo-ku, Tokyo 113-8421, Japan; 2 Intractable Disease Research Center, Graduate School of Medicine, Juntendo University, 2-1-1, Hongo, Bunkyo-ku, Tokyo 113-8421, Japan; 3 Center for Genomic and Regenerative Medicine, Graduate School of Medicine, Juntendo University, 2-1-1, Hongo, Bunkyo-ku, Tokyo 113-8421, Japan; 4 Department of Plastic and Reconstructive Surgery, School of Medicine, Juntendo University, 2-1-1, Hongo, Bunkyo-ku, Tokyo 113-8421, Japan

**Keywords:** diabetes mellitus, wound healing, mononuclear cell therapy, peripheral blood mononuclear cells, quantity and quality control culture technique, chronic foot ulcer, fibroblast migration, PDGF B

## Abstract

A major symptom of diabetes mellitus (DM) is unfit hyperglycemia, which leads to impaired wound healing. It has been reported that the migration of fibroblasts can be suppressed under high glucose (HG) conditions. In our previous study, we introduced a serum-free culture method for mononuclear cells (MNCs) called quantity and quality control culture (QQc), which could improve the vasculogenic and tissue regeneration ability of MNCs. In this study, we described a culture model in which we applied a high glucose condition in human dermal fibroblasts to simulate the hyperglycemia condition in diabetic patients. MNC-QQ cells were cocultured with fibroblasts in this model to evaluate its role in improving fibroblasts dysfunction induced by HG and investigate its molecular mechanism. It was proven in this study that the impaired migration of fibroblasts induced by high glucose could be remarkably enhanced by coculture with MNC-QQ cells. PDGF B is known to play important roles in fibroblasts migration. Quantitative PCR revealed that MNC-QQ cells enhanced the gene expressions of PDGF B in fibroblasts under HG. Taken with these results, our data suggested a possibility that MNC-QQ cells accelerate wound healing via improving the fibroblasts migration and promote the gene expressions of PDGF B under diabetic conditions.

## 1. Introduction

Diabetes mellitus (DM) is one of the most significant diseases that affects more than 415 million people worldwide, and it is estimated that the number of people suffering from this disease will surge to 640 million by 2040 [1]. A major symptom of DM is unfit hyperglycemia, which can lead to severe complications in diabetic patients. One of the complications is chronic nonhealing wound healing, which can result in limb amputations if left untreated [1,2]. The 5-year mortality rates after lower extremity amputation range from 39% to 68% [3]; therefore, developing a new lower limb-conserving therapy for chronic diabetic foot ulcers is at an urgent need.

Endothelial progenitor cells (EPCs) are identified as bone marrow (BM)-derived endothelial precursor cells that contribute to neovascularization [4,5]. It has been demonstrated that autologous BM and peripheral blood MNC (PbMNC) therapy with functional EPCs are clinically effective in ischemia and have been used for clinical vascular regenerative therapy successfully [6,7,8,9]. A previous study has also indicated that the autologous transplantation of mobilized peripheral blood CD34+ cells is an effective and safe therapeutic approach for nonhealing chronic diabetic wounds [10]. However, in diabetic patients, the clinical value of autologous EPCs is limited due to its severely impaired number and functional capacity [10,11,12]; hence, an excess of peripheral blood-collecting to isolate a sufficient amount of EPCs since vasculogenic cells, such as CD34+ or CD133+ cells, constitute a very small percentage of PbMNCs (0.01%) and BMMNCs (0.1%) [10], which could be a huge burden for diabetic patients. Therefore, isolation techniques with a simple methodology and minimal effort are needed.

To overcome these limitations, as an innovative strategy for the treatment of diabetic foot ulcers, we have previously established a novel cell named MNCQQ cells, which is from a serum-free quantity and quality control culture (QQc) of PbMNC where not only was the number of proinflammatory and anti-regenerative cells remarkably decreased, but also, the amount of total and differentiated colony-forming EPCs conditioned for anti-inflammatory and regenerative phenotypes was greatly augmented [13,14]. QQc only requires a simple PbMNC isolation technique, and then, MNCQQ cells can be obtained after 7 days of culturing in expansion media containing five types of cytokines. Moreover, QQc only needs a small amount of blood from the patients and a simple cell isolation technique, which makes it much more practical and easier to perform [13]. We have proved that post-QQc-treatment diabetic PbMNC wounds significantly increased the granulation thickness compared to the pre-QQc DM PbMNC group in euglycemic mice [15]. In our clinical study [16], we showed that MNC-QQ cells accelerate vasculogenesis and wound healing in diabetic patients. Wounds transplanted with post-QQc diabetic cells showed significantly more vessel tissues, which suggests promoted high vasculogenic potential induced by MNCQQ cells. Although we have proven that MNCQQ cells could enhance wound healing via mediating the secretion of metallopepidase-9 (MMP-9) [17], wound healing is a rather complicated process involving numerous phases and cell types [18]; therefore, the underlying molecular mechanism of MNCQQ improving wound healing is still not fully elucidated.

In the wound-healing process, a fibroblast is to be known as the most important cell involved in producing and remodeling the extracellular matrix, and fibroblast cell migration plays a key role in the formation of granulation tissue and further wound repair [19,20]. However, it has been reported that fibroblast migration is impaired under a high glucose (HG) condition compared to a normal glucose (NG) condition [18,21,22].

We hypothesized that MNCQQ cells could restore the dysfunction of fibroblasts migration induced by hyperglycemia in diabetic conditions via promoting the expression of wound healing-related growth factors. This study was designed to evaluate the therapeutic effects of MNCQQ cells on fibroblasts under diabetic conditions and elucidate the detailed mechanism, which could contribute to enhance the effect of MNCQQ cells and improve QQc into being a better treatment for patients with diabetic foot ulcers.

## 2. Materials and Methods

### 2.1. Fibroblast Culture

Normal adult human dermal fibroblasts (fibroblasts) were obtained from Lonza (#CC-2511, Walkersville, MD, USA) and then cultured in Dulbecco’s modified Eagle’s medium (DMEM) containing 1 g/L D-glucose (normal glucose, NG, #11885-084 Gibco) or 4.5 g/L D-glucose (high glucose, HG, #11995-065 Gibco), supplemented with 10% fetal bovine serum (FBS) and 1% penicillin in a humidified incubator at 37 °C with 5% CO_2_ in the atmosphere. The medium was replaced every other day. Cells were harvested by –Trypsin-EDTA (#25300-062,Gibco, Waltham, MA, USA) when cell confluence reached approximately 80%. Experiments were performed using cultured cells that underwent six to ten passages.

### 2.2. QQ Culture of PbMNCs

All volunteers gave their written informed consent for inclusion under the institutional approval of Research Ethics Committee, Faculty of Medicine, Juntendo University before they participated in the study. All study procedures were conducted in accordance with the principles of the World Medical Association Declaration of Helsinki. Nine healthy volunteers were recruited for this study.

First, Pb samples were drawn into vacuum blood collection tubes (#362761, BD, Franklin Lakes, NJ, USA) containing sodium citrate by venipuncture at the forearm and then centrifuged (1800× *g*, 25 °C, 20 min). The PbMNCs in the buffy coat layer were collected and suspended in PBS (#T900, Takara, Kusatsu, Shiga, Japan) containing 2 mM EDTA for further centrifugation (300× *g*, 25 °C, 10 min). Then, erythrocytes were eliminated from PbMNCs using ACK Lysing Buffer (#A1049201, Gibco) and centrifuged with PBS twice (200× *g*, 25 °C, 10 min) for washing. Isolated PbMNCs were cultured at the cell density of 2 × 10^6^ cells/2 mL of QQ culture medium per well of 6-well Primaria tissue culture plates (#353846, BD Falcon, Franklin Lakes, NJ, USA). QQ culture medium was prepared from Stem Line II (#S0192, Sigma-Aldrich, Burlington, MA, USA) supplemented with the 5 human recombinant proteins: stem cell factor (SCF); thrombopoietin (TPO); Flt-3 ligand; vascular endothelial growth factor (VEGF); interleukin (IL)-6 and antibiotics (Penicillin at 100 units/mL and Streptomycin at 100 mg/mL, Gibco, NY, USA). After 7 days of incubation at 37 °C with 5% CO_2_ in the atmosphere, MNCQQ cells were harvested by gently pipetting and washing with IMDM (#31980-030, Gibco, Waltham, MA, USA) and PBS-EDTA for further experiments. The contents of the QQ culture medium are listed in Table 1.

### 2.3. Fluorescence Activated Cell Sorting

MNCQQ cells in PBS-EDTA containing 2% FBS as the FACS buffer were treated with FcR-blocking reagent (Miltenyi, Auburn, CA, USA) at 4 °C for 30 min and then stained with proper isotype controls or the specific antibodies listed in Table 2 for each color at 4 °C for 30 min. The cells were then investigated with an FACS Celesta^TM^ Cell Analyzer flow cytometer (BD Bioscience, Franklin Lakes, NJ, USA) after washed twice using the FACS buffer. The data were analyzed using FlowJo software (Tree Star, Ashland, OR, USA). The scattergrams consisted of three gates indicating three different cell sizes, with the smallest cells in gate A identifying the lymphocyte area, the middle cell size in gate B identifying monocytes, and the large cell size in gate C. The absolute cell number was calculated by multiplying the FACS % of each cell type with the total cell number of 1 × 10^7^ cells collected from MNCQQ cells.

### 2.4. Wound-Healing Assay

The migration ability of fibroblasts was assessed using the wound-healing scratch assay. The cells (8 × 10^3^/well) were seeded on a 96-well plate (#4379 ImageLock, Sartorius, Göttingen, Germany) and cultured in DMEM containing 5 mM or 25 mM D-glucose, 0.5% FBS, and 1% penicillin overnight for serum starvation. After the cells attached to the bottom of the flask, the cells were treated with mitomycin C (1 μg/mL) as a cell proliferation inhibitor overnight to eliminate the proliferation of fibroblasts, and then, a linear scratch was made by scraping across the cell monolayer in each well with sterile pipette tips (200 μL). After the floating cell debris was removed by washing with PBS, the cells were treated with or without the PDGF B inhibitor AG 1296 (AG, #658551, 1 μM Sigma-Aldrich, Burlington, MA, USA) and cocultured with or without MNCQQ cells (8 × 10^3^/insert) or MNC (cell number/insert) in cell culture inserts (0.4 mm, #7369, Corning, New York, NY, USA) for 24 h. Two types of cells were cocultured in a noncontact manner using the culture insert device. An inverted phase-contrast microscope was used to photograph the cells for further analysis. The area denuded of cells was photographed using an inverted phase-contrast microscope and quantified with ImageJ software. The ratio of the original area denuded of cells to that covered with cells after 0–24 h of treatment was used as the data to indicate the degree of migration situation.

### 2.5. qRT-PCR

Fibroblasts (2 × 10^5^/well) were seeded on a 6-well plate (#353502 Multiwell 6 well, Falcon, New York, NY, USA), cultured overnight, and the linear scratch was made as described above. After that, fibroblasts were cocultured with or without MNCQQ cells (2 × 10^5^/well) by using cell culture inserts (#353090, Falcon, New York, NY, USA) for three hours. Fibroblast samples were then collected by gently scraping the cells off the bottom of the flask into the buffer RLT (#79216, Qiagen, Hilden · Düsseldorf, Germany) containing 10% of 2-Mercaptoethanol (#131-14572, Wako, Midtown West, Tokyo Midtown · Akasaka, Minato, Tokyo, Japan ) with a cell scraper (#9000-220, IWAKI). Total RNA was extracted from the fibroblast samples using a RNeasy Micro Kit (#74004, Qiagen, Hilden · Düsseldorf, Germany). RNA concentrations were quantified by NanoDrop One (Thermo Fisher Scientific, Waltham, MA, USA). cDNA was synthesized using a High-Capacity cDNA Reverse Transcription Kit with a RNase Inhibitor (#4374966, Thermo Fisher Scientific, Waltham, MA, USA). Real-time qRT-PCR was performed using the PowerUp SYBR Green Master Mix (#A25742, Thermo Fisher Scientific, Waltham, MA, USA) on a Step One Plus PCR system (Applied Biosystems, Waltham, MA, USA). The primer pairs used for the qRT-PCR analysis are in Table 3. The relative mRNA expression was calculated by using the ΔΔCt method with normalization against human GAPDH (hGAPDH).

### 2.6. Statistical Analysis

All data were expressed as the mean ±standard error. The statistical significance between two groups was analyzed by the Student’s *t*-test. For all tests, *p* < 0.05 was considered statistically significant.

## 3. Results

### 3.1. Positive Cell Population in MNCQQ Cells

**Figure 1:**
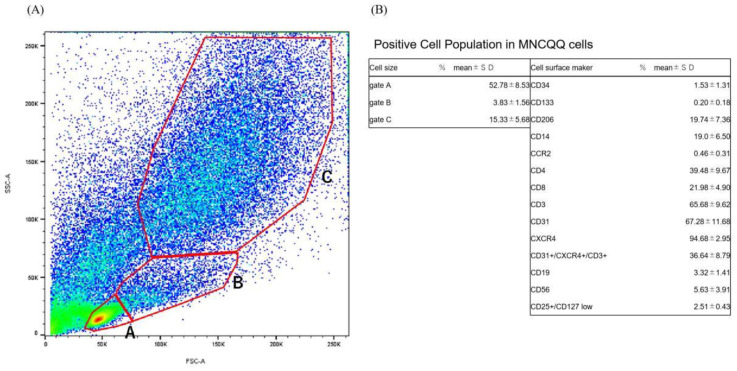
Flow cytometry analysis of MNCQQ cells. (**A**). Scatter diagrams of MNCQQ cells in flow cytometry. The red lines indicate the cellular-sized gates of lymphocyte (A), monocyte (B), or the larger cell (C). (**B**). The table shows cell positivity in MNCQQ cells. *N* = 5 volunteers. The investigated cell surface markers were as follows: hematopoietic stem cell (CD34 and CD133); M2 macrophage (CD206); monocyte (CD14); M1 macrophage (CCR2); endothelial cell (CD31); T cell (CD4, CD8, CD3, CD3/CD31/CXCR4, and CD25+/CD127 low); B cell (CD19); and NK cell (CD56).

Fluorescent cell sorting was carried out to investigate the cell population of MNCQQ cells. Based on the results, MNCQQ cells exhibited a richness in the large cells population (Figure 1A). The proportion of each positive cell involved in the whole cells of the (a), (b), and (c) gates separated with red lines was then estimated.

First of all, MNCQQ cells showed a positivity of CD34+ (1.53 ± 1.31%) and CD133+ (0.20 ± 0.18%) stem cell populations. The anti-inflammatory M2-type (CD206^+^) cells were also detected in MNCQQ cells (19.74 ± 7.36%). In contrast, the cell population of proinflammatory M1-type (CCR2^+^) cells showed a relatively weak positivity (0.46 ± 0.31%) compared to CD206 cells. A similar weak cell positivity was also seen in B-lymphoid cells (CD19^+^, 3.32 ± 1.41%) and natural killer (NK) cells (CD56^+^, 5.63 ± 3.91%). In the T-lymphoid cell population, T-helper cells (CD4^+^, 39.48 ± 9.67%), T-killer cells (CD8^+^, 21.98 ± 4.90%), T-cell coreceptor (CD3^+^, 65.68 ± 9.62%), and endothelial cells (CD31^+^, 67.28 ± 11.68%) constituted the largest proportions of cell populations in MNCQQ cells. Notably, angiogenic T cells (CXCR4^+^/CD31^+^/CD3^+^), which are known to constitute the center of EPC colonies during cultures of human peripheral blood mononuclear cells, also [23] showed great positivity in MNCQQ cells (36.64 ± 8.79%).

These data indicate that MNCQQ cells are a cluster of cells that consist of stem/progenitor cell populations of EPCs, as well as anti-inflammatory and angiogenic monocytes/T-lymphocytes.

### 3.2. MNCQQ Promoted the Migration under Both NG and HG Conditions

To determine the effects of MNCQQ cells on wound healing, the migration of fibroblasts cocultured with or without MNCQQ cells was examined by the wound-healing assay.

**Figure 2:**
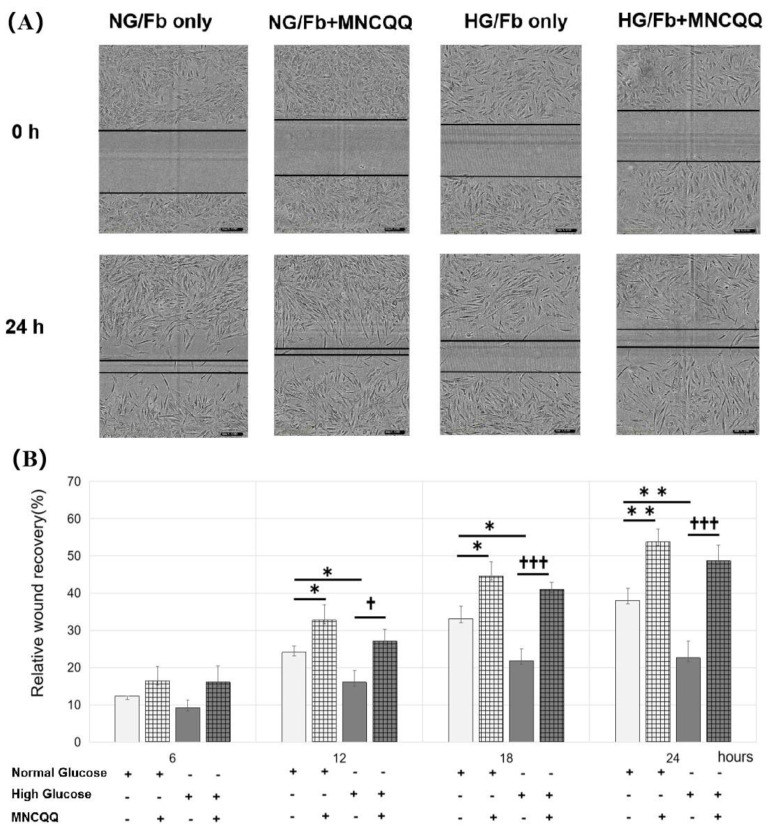
Effect of MNCQQ cells on fibroblasts migration under NG and HG conditions. (**A**) Migration ability was measured by the wound−healing assay, and (**B**) the relative wound recovery was presented as % recovery of the wound distance at 6, 12, 18, and 24 h relative to 0 h (magnification, ×100). *N* = 3 volunteers. *; vs. NG/Fb only, *p* < 0.05, **; vs. NG/Fb only, *p* < 0.01, †; vs. HG/Fb only, *p* < 0.05, †††; vs. HG/Fb only, *p* < 0.005. NG/Fb only: fibroblasts only in normal glucose; HG/Fb only: fibroblasts only in high glucose; NG/Fb + MNCQQ: fibroblasts cocultured with MNCQQ cells in normal glucose; HG/Fb + MNCQQ: fibroblasts cocultured with MNCQQ cells in high glucose.

The results demonstrated that, although the migration of fibroblasts was impaired under the HG condition compared to the NG condition (38.1 ± 10.2% vs. 22.6 ± 6.9%/24 h; *p* < 0.01), the migration of fibroblasts cocultured with MNCQQ cells was significantly promoted compared to those without a coculture not only under NG (38.1 ± 10.2% vs. 53.7% ± 9.0%/24 h; *p* < 0.01) but also HG conditions (22.6 ± 6.9% vs. 48.7 ± 8.0%/24 h; *p* < 0.005) (Figure 2). Taken together, these results indicated that MNCQQ cells increased the impaired migration of fibroblasts caused by HG.

### 3.3. Platelet-Derived Growth Factor (PDGF) B in Fibroblasts Were Upregulated after Coculture with MNCQQ Cells

**Figure 3:**
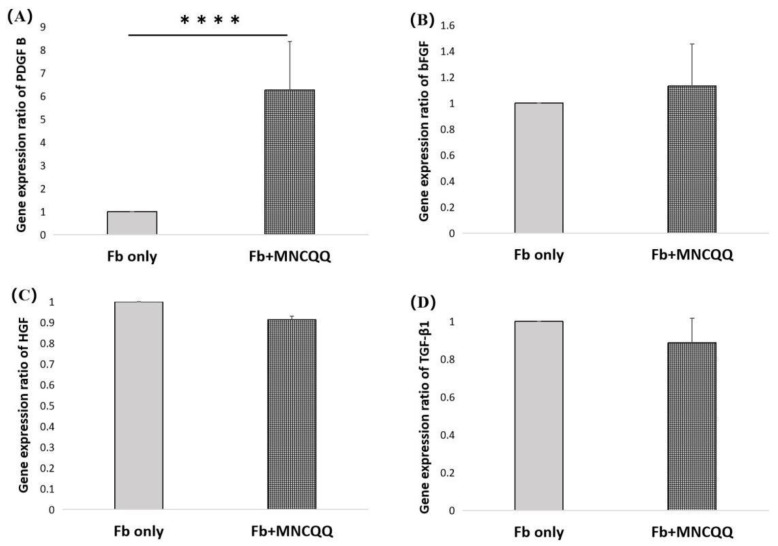
mRNA expression in fibroblasts under the HG condition. Reverse transcription-quantitative polymerase chain reaction for the mRNA expression levels of PDGF B (**A**), bFGF (**B**), HGF (**C**), and TGF-β1 (**D**), respectively, in fibroblast cells in the HG condition. *N* = 6 volunteers. ****; vs. Fb only, *p* < 0.001 Fb only: fibroblasts only in high glucose; Fb + MNCQQ: fibroblasts cocultured with MNCQQ cells in high glucose.

In order to analysis the potential mechanisms underlying the effects of MNCQQ cells on the migration of fibroblasts under HG conditions, the expressions of genes related to wound-healing, including PDGF B, basic Fibroblast Growth Factor (bFGF), Hepatocyte Growth Factor (HGF), and Transforming growth factor (TGF)-β1, were examined using RT-qPCR. The results showed that only the expressions of PDGF-B were significantly upregulated in fibroblasts and MNCQQ cells cocultured group in HG conditions (1.8 ± 1.0 vs. 8.9 ± 5.1; *p* < 0.05) (Figure 3).

### 3.4. The Promoted Migration and PDGF B Expression of Fibroblasts Induced by MNCQQ Cells Was Impressed by PDGFR Inhibitor (AG)

**Figure 4:**
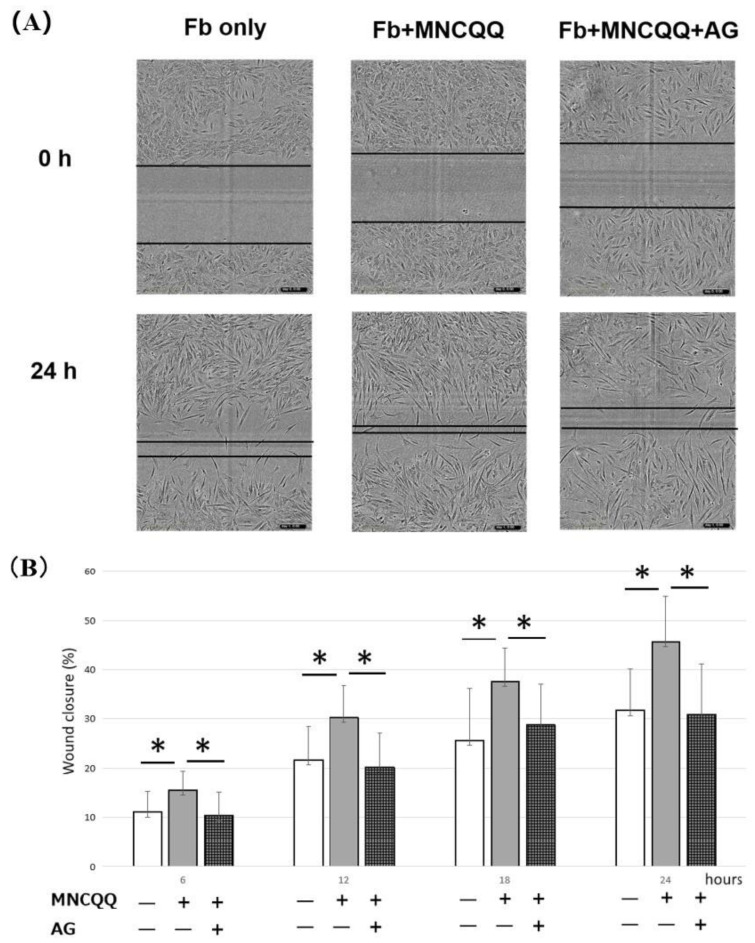
Effect of MNCQQ cells on fibroblasts migration under HG conditions. (**A**) Migration ability was measured by the wound−healing assay, and (**B**) the relative wound recovery was presented as % recovery of the wound distance at 6, 12, 18, and 24 h relative to 0 h (magnification, ×100). *N* = 4 volunteers. *; vs. Fb + MNCQQ, *p* < 0.05; Fb only: fibroblasts only in high glucose; Fb + MNCQQ: fibroblasts cocultured with MNCQQ cells in high glucose; Fb + MNCQQ + AG: fibroblasts cocultured with MNCQQ cells in high glucose with AG applied.

**Figure 5:**
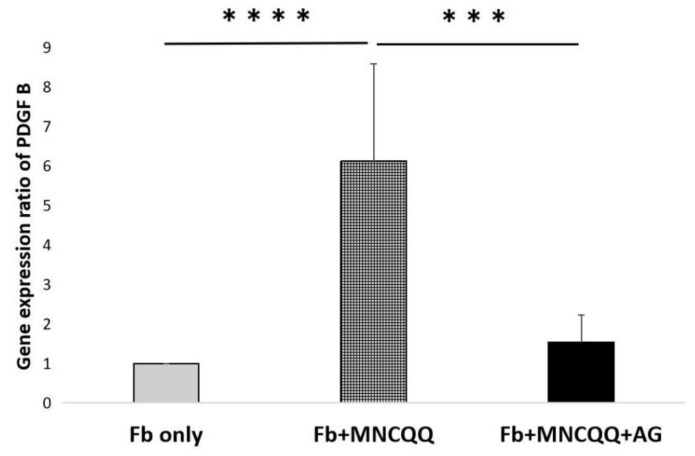
mRNA expression of PDGF B in fibroblasts under HG condition. Reverse transcription-quantitative polymerase chain reaction for the mRNA expression levels of PDGF B in fibroblast cells in the HG condition. *N* = 7 volunteers. ***; vs. Fb + MNCQQ, *p* < 0.005; ****; vs. Fb + MNCQQ, *p* < 0.001 Fb only: fibroblasts only in high glucose; Fb + MNCQQ: fibroblasts cocultured with MNCQQ cells in high glucose; Fb + MNCQQ + AG: fibroblasts cocultured with MNCQQ cells with AG applied in high glucose.

The PDGF/PDGF receptor (PDGFR) has been shown to be associated with the migration of fibroblasts. Therefore, a PDGFR inhibitor was applied to determine if the migration ability and the mRNA expressions of PDGF-B in fibroblasts cocultured with MNCQQ cells can be influenced by PDGFR. We examined the migration of fibroblasts cocultured with or without MNCQQ cells in the presence of AG using the wound-healing assay and the mRNA expression of PDGF B in fibroblasts using RT-qPCR. The results from the wound-healing assay demonstrated that the migration ability of fibroblasts cocultured with MNCQQ cells in the presence of AG was downregulated compared to the fibroblasts cocultured with MNCQQ cells without the AG (38.1 ± 10.2% vs. 30.8 ± 10.2% /24 h; *p* < 0.01) (Figure 4). RT-qPCR also showed the same downregulation of PDGF B mRNA expression in fibroblasts cocultured with MNCQQ cells in the presence of AG (9.3 ± 5.0 vs. 2.7 ± 1.2; *p* < 0.05) (Figure 5).

These results suggest that MNCQQ cells might regulate the migration of fibroblasts though the PDGF/PDGFR signaling pathway.

## 4. Discussion

The migration of dermal fibroblasts is essential for cutaneous wound repair, because dermal fibroblasts migrate to damaged sites, repopulate the wound, and remodel fibrin and collagen deposits [24].However, accumulating evidence has revealed that wound healing can be impaired by hyperglycemia condition in diabetic patients due to poor fibroblasts migration [18,25].We previously demonstrated that MNCQQ cell therapy could enhance wound closure, maturation, and vascularization in diabetic wounds suggesting that MNCQQ cells could improve wound healing [13,14,15].In this study, we hypothesized that MNCQQ cells could restore the impaired migration of fibroblasts induced by HG environment in diabetic condition. Our results showed that not only can MNCQQ cells promote the migration of fibroblasts under normal glucose condition, but also under high glucose condition which simulate the hyperglycemia condition in diabetic patients. 

Our previous research has confirmed mRNA levels changes in the expression of genes related to angiogenesis, wound-healing, and anti-inflammation between pre- and post-QQ cells in patients with DM. The gene expression levels of many pro-angiogenic growth factors as well as cytokines and wound-healing genes in DM PbMNC were significantly increased post-QQc treatment including, PDGF B, IGF-1, IL-6 and TGF-β [15]. It has been reported that stimuli from these promoted genes can induce wound healing related growth factors including PDGF in various types of cells such as endothelial cells, smooth muscle cells or fibroblasts [26,27,28,29].

Wound healing is a complex process and can be regulated by numerous growth factors, and it has been showed that the depression of their expressions may lead to impaired wound healing process [30]. The potent mitogen, PDGF, is a strong class of fibroblasts stimulating factors [31] and has five isoforms, PDGF-AA, PDGF-AB, PDGF-BB, PDGF-CC and PDGF-DD, built up of four different polypeptide chains encoded by four different genes [32,33]. Of these isoforms, PDGF BB which is built up by PDGF B could accelerate wound healing possibly via promoting the deposition of extracellular matrix, angiogenesis and the migration of fibroblasts [34,35].Furthermore, it has been reported that PDGF can improve wound healing in diabetic condition and help chronic ulcers heal faster especially with the application of high doses of recombinant PDGF-BB [36]. However, we found out that prior to QQc, the expression of PDGF was lower in DM PbMNCs compared to those in healthy controls [15], and other previous study has confirmed that only little PDGF can be detected in chronic nonhealing wounds [37]. To elucidate the mechanism underlying the therapeutic effect of MNCQQ on the migration of fibroblasts under high glucose condition, we investigated the expression of PDGF B and other wound-healing related growth factors in fibroblasts after cocultured with MNCQQ cells.

Real time PCR results show that MNCQQ cells can promote the expressions of PDGF B in human dermal fibroblasts suggesting that the improved migration of fibroblasts induced by MNCQQ cells may be related to PDGF B’s promotion which is possibly induced by stimuli of secreted growth factors or cytokines from MNCQQ cells. 

PDGFs functionalize by binding PDGFRs to activate these receptors dimerize and downstream signal transduction, for example, through the PI3K pathway or through reactive oxygen species (ROS)-mediated activation of the STAT3 pathway [38]. It is known that PDGFR β binds with high affinity to PDGF-BB and the PDGF BB/PDGFR β/PI3K signaling pathway has been reported to be associated with the migration of fibroblasts [34]. In this study PDGFR inhibitor was applied to evaluate if the migration ability and the mRNA expression of PDGF-B in fibroblasts cocultured with MNCQQ cells is influenced by PDGFR. The results showed that the migration ability and the expression of PDGFs were downregulated when AG was applied suggesting MNCQQ cells might regulate the migration of fibroblasts though the PDGF/PDGFR signaling pathway.

Although our results suggest that it is possible that MNCQQ cells accelerate wound healing via improving the fibroblasts migration by promoting the gene expressions of PDGFs of fibroblasts under diabetic condition, there are still many places in this research that need further investigations. For example, we could detect the expressions of PDGF’s downstream pathways such as PI3K/Akt to further clarify the mechanism of MNCQQ cells. In addition, we can also silence the PDGFR gene to further evaluate PDGF’s influence on migration of fibroblasts cocultured with MNCQQ cells. Wound healing is not only affected by the migration of fibroblasts but a complex involving highly orchestrated phases and factors. Future studies will need to explore other pathways and growth factors to further clarify the effect and mechanism of MNCQQ cells on wound healing.

QQc is the world’s first cell therapy for improving vasculogenesis and wound healing that can be performed only by collecting blood. Elucidating the detailed mechanism of the therapeutic effect of MNCQQ cells can contribute to progress QQc into a simpler, safer, and more effective regenerative therapy for not only diabetic patients but also more populations of patients. Therefore, it’s academic value is significant.

## Figures and Tables

**Table 1:** Contents of the QQ Culture Medium.

	Company, Catalog No.	Final Concentration
StemlineⅡTM Hematopoietic Sten Cell Expansion Medium	Sigma-Aldrich, #S0192	
rh SCF	Peprotec #300-07	100 ng/mL
rh Flt-3 ligand	Peprotec #300-19	100 ng/mL
rh TPO	Peprotec #300-18	20 ng/mL
rh VEGF	Peprotec #100-20	50 ng/mL
rh IL-6	Peprotec #200-06	20 ng/mL

rh, recombinant human.

**Table 2:** Antibodies for flow cytometry.

Antibody	Catalog No.	Company
PE/Cy 7-labeled anti-CD 31	clone: WM59	Biolegend, San Diego, California
PE-labeled anti-CD 34	clone: 581	Biolegend
APC-labeled anti-CD 184 (CXCR 4)	clone: 12G5	Biolegend
APC-labeled anti-CD 133	clone: AC133	Miltenyi, Bergisch Gladbach, North Rhine-Westphalia, Germany
Alexa Fluor-700-labeled anti-CD 3+	clone: UCHT1	Biolegend
APC-Cy 7-labeled anti-CD 14	clone: HCD14	Biolegend
PE/Cy 7-labeled anti-CD 206 (MMR)	clone: 15-2	Biolegend
PerCP/Cy 5.5-labeled anti-CCR 2 (CD 192)	clone: K036C2	Biolegend
BV421-labeled anti-CD 127	clone: A019D5	Biolegend
BV421-labeled anti-CD 8	clone: RPA-T8	Biolegend
FITC-labeled anti-CD 4	clone: RPA-T4	Biolegend
PE-labeled anti-CD 25	clone: M-A251	Biolegend

**Table 3:** Human PCR primers for qRT-PCR.

Markers	Forward Sequences	Revers Sequences
GAPDH	5′-GGCCTCCAAGGAGTAAGACC-3′	5′-GACTGAGTGTGGCAGGGACT-3′
PDGF-B	5′-TGAGAAAGATCGAGATTGTGCG-3′	5′-GGGCTTCGGGTCACAGG-3′
bFGF	5′-ACCTCACATCAAGCTAC-3′	5′-GTTTCAGTGCCACATACC-3′
HGF	5′-ATGCATCCAAGGTCAAGGAG-3′	5′-TTCCATGTTCTTGTCCCACA-3′
TGF-β1	5′-CCCAGCATCTGCAAAGCTC-3′	5′-GTCAATGTACAGCTGCCCGCA-3′

The primers were synthesized by Thermo Fisher Scientific.

## Data Availability

Not applicable.
